# Potential Association of Gut Microbial Metabolism and Circulating mRNA Based on Multiomics Sequencing Analysis in Fetal Growth Restriction

**DOI:** 10.1155/2024/9986187

**Published:** 2024-04-05

**Authors:** Hui Tang, Dan Li, Jing Peng, Weitao Yang, Xian Zhang, Hanmei Li

**Affiliations:** Hunan Provincial Key Laboratory of Regional Hereditary Birth Defects Prevention and Control, Changsha Hospital for Maternal and Child Health Care Affiliated to Hunan Normal University, Changsha, China

## Abstract

**Objective:**

Fetal growth restriction (FGR) is a significant contributor to negative pregnancy and postnatal developmental outcomes. Currently, the exact pathological mechanism of FGR remains unknown. This study aims to utilize multiomics sequencing technology to investigate potential relationships among mRNA, gut microbiota, and metabolism in order to establish a theoretical foundation for diagnosing and understanding the molecular mechanisms underlying FGR.

**Methods:**

In this study, 11 healthy pregnant women and nine pregnant women with FGR were divided into Control group and FGR group based on the health status. Umbilical cord blood, maternal serum, feces, and placental tissue samples were collected during delivery. RNA sequencing, 16S rRNA sequencing, and metabolomics methods were applied to analyze changes in umbilical cord blood circulating mRNA, fecal microbiota, and metabolites. RT-qPCR, ELISA, or western blot were used to detect the expression of top 5 differential circulating mRNA in neonatal cord blood, maternal serum, or placental tissue samples. Correlation between differential circulating mRNA, microbiota, and metabolites was analyzed by the Spearman coefficient.

**Results:**

The top 5 mRNA genes in FGR were altered with the downregulation of TRIM34, DEFA3, DEFA1B, DEFA1, and QPC, and the upregulation of CHPT1, SMOX, FAM83A, GDF15, and NAPG in newborn umbilical cord blood, maternal serum, and placental tissue. The abundance of *Bacteroides*, *Akkermansia*, *Eubacterium_coprostanoligenes_group*, *Phascolarctobacterium*, *Parasutterella*, *Odoribacter*, *Lachnospiraceae_UCG_010*, and *Dielma* were significantly enriched in the FGR group. Metabolites such as aspartic acid, methionine, alanine, L-tryptophan, 3-methyl-2-oxovalerate, and ketoleucine showed notable functional alterations. Spearman correlation analysis indicated that metabolites like methionine and alanine, microbiota (*Tyzzerella*), and circulating mRNA (TRIM34, SMOX, FAM83A, NAPG) might play a role as mediators in the communication between the gut and circulatory system interaction in FGR.

**Conclusion:**

Metabolites (METHIONINE, alanine) as well as microbiota (*Tyzzerella*) and circulating mRNA (TRIM34, SMOX, FAM83A, NAPG) were possible mediators that communicated the interaction between the gut and circulatory systems in FGR.

## 1. Introduction

Fetal growth restriction (FGR) is a serious complication of pregnancy associated with an increased risk of perinatal mortality and morbidity [1]. Recent studies have shown that twins with selective FGR and altered fecal metabolism display dysbiosis of the gut microbiota, characterized by the decreased abundance of *clostridia* and *bacteroides*, as well as the reduced levels of arginine and homocysteine [2]. Evidence suggests that the maternal gut microbiota plays a critical role in the development of FGR and normal fetal growth [3]. Inadequate maternal nutrition can impact the composition of specific gut microbiota, potentially affecting maternal micronutrient status and fetal development, thereby influencing pregnancy outcomes [4]. These findings highlight a potential association between FGR and the stability of maternal gut microbiota, although the precise mechanisms involved remain unclear.

FGR is also known as intrauterine growth restriction (IUGR) [5, 6]. At the taxonomic level, the prevalence of *Neisseriaceae* in IUGR patients is significantly higher. *Neisseriaceae* is a mucosal *β*-hemolytic bacterium known to absorb iron-binding host proteins, including hemoglobin [7]. The placental microbiome composition may potentially serve as a biomarker for fetal health during pregnancy and offer insights into its role in the development of IUGR [8]. FGR is linked to elevated levels of placental macrophages and proinflammatory markers in both the placenta and maternal serum [9]. Studies in mice have demonstrated that supplementing IUGR mothers with curcumin can improve intestinal integrity, oxidative status, and gut microbiota of male offspring [10]. These findings suggest a relationship between FGR and the maternal placental microenvironment, although the specific mechanisms involved are not yet fully understood.

Research has revealed a close association between FGR and the functionality of trophoblast cells in the placenta [11–15]. An important finding from a clinical trial suggests that dishevelled-associated activator of morphogenesis 2 (DAAM2) is significantly overexpressed in placentas experiencing compromised fetal growth during pregnancy [16]. When DAAM2 was silenced under hypoxic conditions, it led to a decrease in the expression of the prosurvival gene Bcl-2 and the oxidative stress marker NOX4, while increasing the expression of the antioxidant enzyme HMOX-1 [17]. Elevated levels of Bcl-2 in the serum of FGR mothers and in the umbilical cord have been identified as potential perinatal markers for late preterm pregnancies with IUGR [18]. Another clinical trial indicated that FGR contributes to elevated levels of fetal hemoglobin in the placenta, leading to the development of a proinflammatory phenotype in endothelial cells [19]. Currently, our understanding of biomarkers for predicting FGR is still limited, highlighting the need for further research in this area.

Based on the aforementioned information, we reasonably speculate that the interaction between the gut microbiome and the metabolic profile plays a role in FGR. The objective of this study is to identify distinctive gene expression patterns in umbilical cord blood from pregnant women with FGR using RNA sequencing analysis to unveil new biomarkers for this condition. Differential gut microbiota was investigated through high-throughput sequencing of 16S rRNA, while variations in metabolites present in fecal samples were scrutinized using untargeted metabolomics. By conducting Spearman's correlation analysis, we aimed to evaluate the relationships between gut microbiota, metabolites, and differentially expressed genes. Our findings may offer insights into the underlying mechanisms of gut microbiota–metabolism–circulatory system interactions and the potential identification of biomarkers for FGR.

## 2. Materials and Methods

### 2.1. Clinical Baseline Data

The 11 healthy pregnant women and nine pregnant women with FGR, who were admitted and delivered in our hospital from July 2021 to July 2022, were selected as the research subjects. They were divided into the Control group and the FGR group. Basic information such as age, body weight, gestational age, body mass index (BMI), and fasting blood glucose of the pregnant women were recorded (Table 1). The inclusion and exclusion criteria for all pregnant women are as follows [20]. Inclusion criteria: (1) FGR must meet the diagnostic criteria of the “2020 Expert Consensus on FGR,” (2) singleton pregnancy, (3) age between 22 and 35, and (4) healthy pregnant women without complications such as gestational hypertension and gestational diabetes. Exclusion criteria: (1) coexisting severe organ diseases such as heart and lung diseases; (2) coexisting severe infection; (3) coexisting history of significant gastrointestinal surgery; and (4) long-term use of antibiotics, hormones, or previous use of probiotics-related preparations. All subjects collected umbilical cord blood, peripheral blood, placental tissue, and feces from pregnant women during childbirth, which were, respectively, used for RNA sequencing (three samples/group), RT-qPCR, ELISA, and western blot testing (five samples/group), as well as fecal microbiome 16S rRNA sequencing and metabolomics analysis (eight samples/group).

### 2.2. RNA Sequencing

The RNA from umbilical cord blood samples was extracted using the DP424 kit (TIANGEN, China). Gel electrophoresis with agarose was performed to analyze the integrity of the RNA samples and check for DNA contamination. Amplification was carried out using Oligo-dT primers. Library preparation steps were followed as instructed in the APExBIO kit (Cat. No. K1159, USA). AMPureXP beads were utilized to isolate cDNA fragments ranging from 250 to 300 bp and to generate purified libraries. Following library construction, an initial quantification was carried out using the Qubit 2.0 Fluorometer. The libraries were then diluted to a concentration of 1.5 ng/*μ*L. Subsequently, library fragment analysis was performed using the Agilent 2200 Bioanalyzer. Once the fragment lengths met the anticipated criteria, sequencing was executed on the Illumina Novaseq 6000 platform. Paired-end sequencing with a read length of 150 bp was conducted on the quality-checked libraries, considering the effective concentration and the desired data volume. Fastp was employed to filter the raw sequencing data (PFdata), eliminating low-quality data and adapters. HISAT2 (v2.1.0) was used to align the sequencing reads to the reference genome hg19 (human) through the annotation file gencode.v34lift37.annotation.gtf (human) [21]. Bam files of the aligned sequences were generated to the reference genome. StringTie was used to assemble and quantify gene abundance [22]. Principal component analysis (PCA) was performed on the samples using the R prcomp function. Differential analysis was performed using the DESeq2. The criteria for differential gene selection were |log2FC| ≥ 1 and *P*-adjust ≤ 0.05.

### 2.3. 16S rRNA Sequencing

According to the manufacturer's instructions, the fecal genome DNA extraction kit (CAT.#DP328-02, TIANGEN, China) was used to extract DNA from fecal samples. Qubit with dsDNA HS Assay Kit (CAT.12640ES76, YEASEN, China) was used for concentration detection. PCR amplification was performed using Phusion enzyme (K1031, APExBIO, USA) and bacterial primers targeting the V3-V4 region of the 16S rRNA gene (357F 5′-ACTCCTACGGRAGGCAGCAG-3′ and 806R 5′-GGACTACHVGGGTWTCTCATAT-3′), followed by adapter and library construction. The Illumina NovaSeq 6000 PE250 platform was utilized for multiplexed sequencing to generate raw sequencing data. The raw data were subjected to quality control using Qiime 2 (2020.2) analysis pipeline and DADA2 to obtain clean data. Species annotation of each ASV/OTU sequence for species abundance was conducted using the Silva-132-99 database. Qiime 2 software was employed to calculate the alpha diversity index for each sample, while the LDA effect size (LefSe, https://github.com/SegataLab/lefse) was used to assess the enrichment of functional pathways for intergroup differences in the analysis.

### 2.4. Metabolomics Analysis

Metabolomics analysis was performed using an UHPLC system (1,290, Agilent Technologies, USA) connected to a UPLC BEH Amide column (1.7 *μ*m 2.1 × 100 mm, Waters, USA), and coupled to both TripleTOF 6600 (Q-TOF, AB Sciex, USA) and QTOF 6550 (Agilent, USA). The R package XCMS (Version 3.2) was used to process the MS raw data files. The preprocessing results generated a data matrix containing retention time (RT), mass-to-charge ratio (m/z) values, and peak intensity. The R package CAMERA was used for peak annotation. Bioinformatics analysis was performed using the MetaboAnalyst platform (https://www.metaboanalyst.ca/) [23]. Metabolite functional prediction was performed using the Kyoto Encyclopedia of Genes and Genomes (KEGG) pathway database (https://www.kegg.jp/).

### 2.5. Reverse Transcription-Quantitative Polymerase Chain Reaction (RT-qPCR)

Trizol (15596026, Thermo, USA) was used to extract total RNA from the samples. The mRNA reverse transcription kit (CW2569, Beijing Kangwei Century, China) was used to synthesize cDNA. The expression of the target gene was detected using the UltraSYBR Mixture kit (CW2601, Beijing Kangwei Century, China) and a fluorescence quantitative PCR instrument (PIKOREAL96, Thermo, USA). The expression of the target gene was calculated using the 2^−*ΔΔ*Ct^ method (Table 2).

### 2.6. Western Blot

Placental tissue was treated with 300 *μ*L of RIPA lysis buffer (AWB0136, Abiowell, China) to extract total protein. Subsequently, 200 *μ*g protein samples were separated using 12% sodium dodecyl sulfate-polyacrylamide gel electrophoresis (SDS-PAGE). The separated proteins were then transferred onto a polyvinylidene fluoride membrane that had been pretreated with methanol and blocked with 5% skim milk (AWB0004, Abiowell, China). Following this, the membrane was incubated overnight at 4°C with the primary antibodies, which included anti-TRIM34 (1 : 5,000, ab180130, Abcam, UK), anti-DEFA3 (1 : 1,000, orb539158, Biorbyt, UK), anti-DEFA1B (1 : 500, 18057-1-AP, Proteintech, USA), anti-DEFA1 (1 : 500, 18057-1-AP, Proteintech, USA), anti-QPCT (1 : 1,000, ab201172, Abcam, UK), anti-CHPT1 (1 : 1,000, ab97672, Abcam, UK), anti-SMOX (1 : 5,000, 15052-1-AP, Proteintech, USA), anti-FAM83A (1 : 1,000, ab259949, Abcam, UK), anti-GDF15 (1 : 2,000, 27455-1-AP, Proteintech, USA), anti-NAPG (1 : 1,000, bs-19016R, Bioss, China), and anti-*β*-actin (1 : 5,000, 66009-1-Ig, Proteintech, USA). Subsequently, the membrane was exposed to secondary antibodies, which included antimouse IgG (1 : 5,000, SA00001-1, Proteintech, USA) and antirabbit IgG (1 : 6,000, SA00001-2, Proteintech, USA), at 37°C for 90 min. Finally, the membrane was visualized using ECL Plus supersensitive luminescent liquid (AWB0005, Abiowell, China) and analyzed with a chemiluminescence imaging system (ChemiScope6100, CLINX, China).

### 2.7. Enzyme-Linked Immunosorbent Assay (ELISA)

Whole blood samples should be kept at room temperature for 2 hr, then centrifuged at 1,000 g for 15 min at 2−8°C. The supernatant can be taken for immediate testing. DEFA1 (CSB-E14155h, CUSABIO, China), QPCT (CSB-EL019135HU, CUSABIO, China), GDF15 (CSB-E12009h, CUSABIO, China), DEFA3 (CSB-EL006655HU, CUSABIO, China), DEFa1B (SEE136Hu, Cloud-Clone Corp, USA), and TRIM3 (SEC224Hu, Cloud-Clone Corp, USA) kits were used for detection. Each well was incubated at 37°C for 2 hr after adding 100 *μ*L of standard or test sample and thoroughly mixing. Following this, 100 *μ*L of biotin-labeled antibody working solution was added to each well and incubated at 37°C for 1 hr. The plate was then washed three times with 200 *μ*L of solution per well for 2 min. Subsequently, 100 *μ*L of horseradish peroxidase-labeled avidin working solution was added to each well and incubated at 37°C for 1 hr. The plate was washed five times before adding 90 *μ*L of substrate solution to each well. The plate was then incubated at 37°C, avoiding light, for 15–30 min. To terminate the reaction, 50 *μ*L of stop solution was added. The optical density (OD) value of each well was promptly measured at a wavelength of 450 nm using an enzyme-linked immunosorbent assay reader (MB-530, HEALES, China) within 5 min after stopping the reaction.

### 2.8. Correlation Analysis

Spearman correlation coefficient was used to analyze the correlation between differential genes (TRIM34, DEFA3, DEFA1B, DEFA1, QPCT, CHPT1, SMOX, FAM83A, GDF15, NAPG) with differential microbiota and metabolites. *P*-value < 0.05 was considered as significant correlation.

### 2.9. Data Statistics and Analysis

The statistical analysis for this research was conducted using GraphPad Prism 8.0 software. Continuous data were presented as mean ± standard deviation. Initially, tests were performed to check for normality and homogeneity of variance. Between-group comparisons were analyzed using nonpaired *t*-tests. Multiple group comparisons were done using one-way analysis of variance (ANOVA) and post-hoc testing was conducted using Tukey's method. A *P*-value of <0.05 was considered statistically significant.

## 3. Results

### 3.1. Differential Expression of mRNA in Cord Blood Circulation

**Figure 1:**
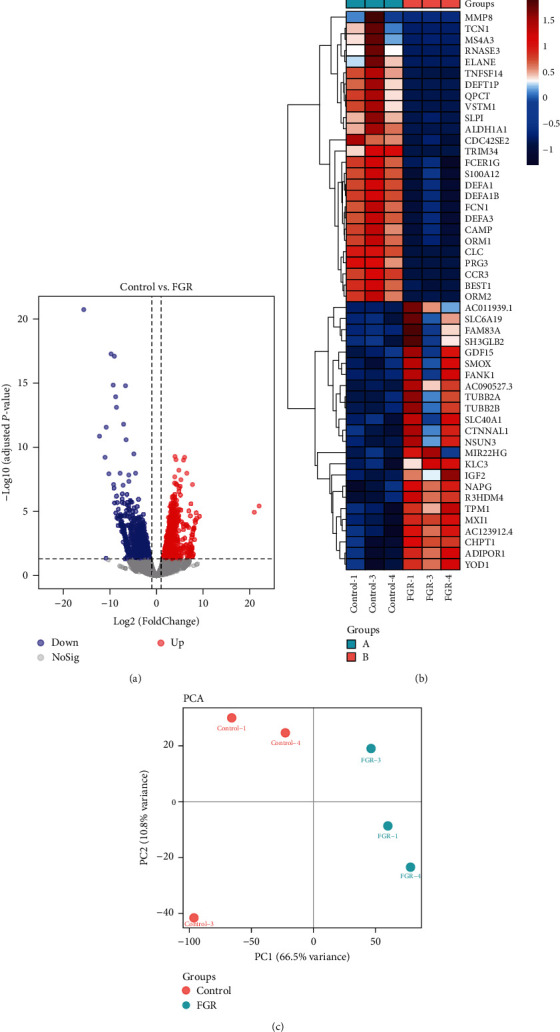
Differentially expression of genes in cord blood circulation: (a) volcano plot showed the differentially expressed genes (DEGs) and (b) heatmap showed top 50 DEGs. Each column and row corresponded to a sample and a mRNA, respectively. Red meant upregulation while blue meant downregulation; (c) PCA analysis. *N* = 3 samples/group in the RNA sequencing.

Umbilical cord blood samples were collected from different groups of pregnant women to analyze the differentially expressed mRNA using RNA sequencing. The volcano plot showed a total of 2202 genes with significant changes in expression, including 1,011 downregulated genes and 1,191 upregulated genes (Figure 1(a)). A heatmap displayed the top 50 genes with differential expression (Figure 1(b)). PCA analysis indicated a significant difference between the Control and FGR groups with PC1 accounting for 66.5% of the variance (Figure 1(c)). These results demonstrated that the mRNA gene expression profile of umbilical cord blood was altered in FGR pregnant women, possibly belong to FGR-related pathogenic genes.

### 3.2. Validation of Differential Expression of mRNAs in Circulation and Placental Tissues

**Figure 2:**
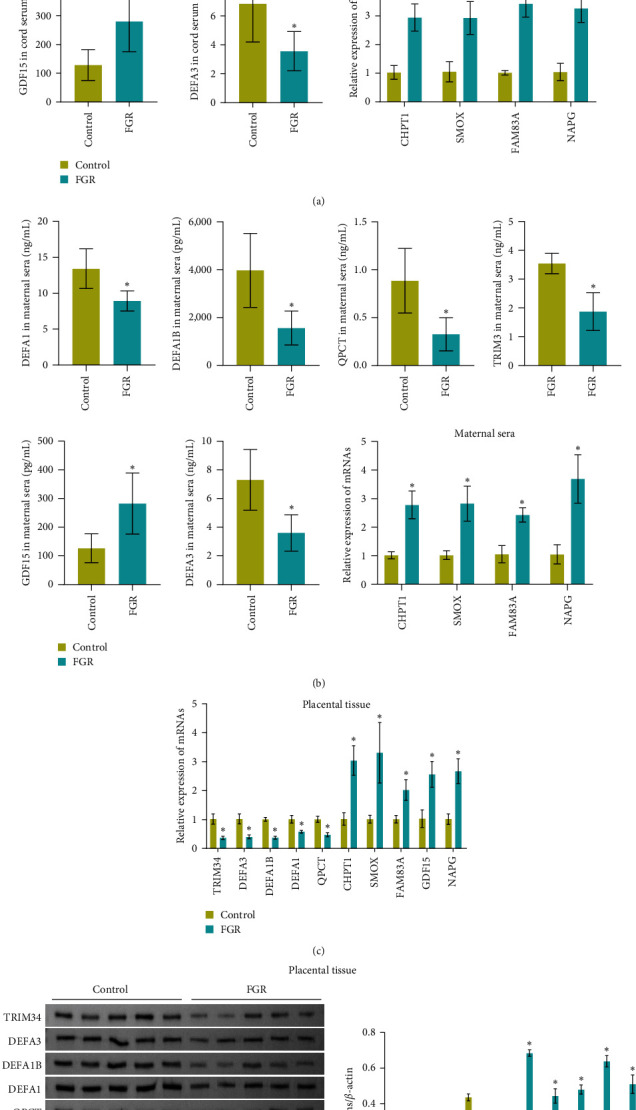
Validation of DEGs in circulation and placental tissues: (a–c) PCR and ELISA were used to detect Top 5 downregulated genes (TRIM34, DEFA3, DEFA1B, DEFA1, QPCT) and Top 5 upregulated genes (CHPT1, SMOX, FAM83A, GDF15, NAPG) in cord serum (a), maternal serum (b), and placental tissue (c). (d) The protein levels of Top 5 downregulated genes (TRIM34, DEFA3, DEFA1B, DEFA1, QPCT) and Top 5 upregulated genes (CHPT1, SMOX, FAM83A, GDF15, NAPG) were detected by western blot in placental tissues.  ^*∗*^*P* < 0.05 vs. control. *N* = 5 samples/group for the validation of DEGs.

Next, we validated the expression of top 5 upregulation and downregulation mRNAs. The results showed that compared with the Control group, the expression of TRIM34, DEFA3, DEFA1B, DEFA1, and QPCT was significantly decreased in cord serum, maternal serum, and placental tissue of FGR group (Figure 2(a)–2(c)). In addition, the expression of CHPT1, SMOX, FAM83A, GDF15, and NAPG was significantly increased in cord serum, maternal serum, and placental tissue of FGR group (Figure 2(a)−2(c)). Protein level detection showed that the expression of TRIM34, DEFA3, DEFA1B, DEFA1, and QPCT was significantly decreased in maternal placental tissue of FGR group (Figure 2(d)). The expression of CHPT1, SMOX, FAM83A, GDF15, and NAPG was significantly increased in maternal placental tissue of FGR group (Figure 2(d)). These results demonstrated that the protein level changes in top 5 upregulation and downregulation genes in maternal circulation and FGR maternal placental tissue might be related to FGR.

### 3.3. Differential Changes of Gut Microbiota in Pregnant Women with FGR

**Figure 3:**
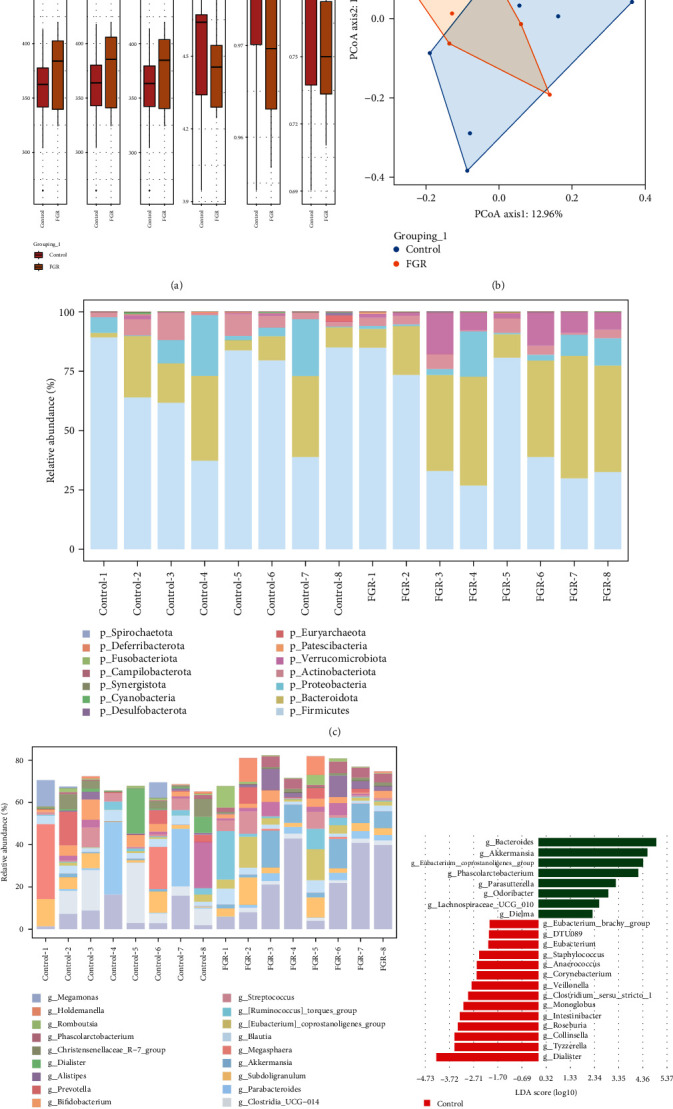
Differential changes of gut microbiota in pregnant women with FGR: (a) alpha index analysis, (b) PCoA analysis, (c, d) microbiota abundance at phylum and genus levels, and (e) LEfSe analysis of difference microbiota at the genus level. *N* = 8 samples/group for the 16S rRNA sequencing.

Fecal specimens from various groups of pregnant women were collected and used to detect differences in intestinal microbiota by high-throughput sequencing of 16S rRNA. Alpha diversity index analysis showed a slight increase in the Observe, Chao1, and ACE indices of fecal microbiota in the FGR group, which indicated an increase in the observed microbial population, microbial, and community richness (Figure 3(a)). However, the Shannon, Simpson, and *J* indices showed a slight decrease in the FGR group, which indicated a decrease in microbial diversity and that no single species was overly dominant in the community (Figure 3(a)). PCoA analysis showed both overlap and separation between the control and FGR groups, indicating intergroup differences (Figure 3(b)). Bar charts further displayed the enrichment of major microbial taxa at the phylum and genus levels (Figure 3(c)–3(d)). Lefse analysis demonstrated that *Bacteroides*, *Akkermansia*, *Eubacterium_coprostanoligenes_group*, *Phascolarctobacterium*, *Parasutterella*, *Odoribacter*, *Lachnospiraceae_UCG_010*, and *Dielma* were significantly increased in abundance in the FGR group. Conversely, *Dialister*, *Tyzzerella*, *Collinsella*, *Roseburia*, *Intestinibacter*, *Monoglobus*, *Clostridium_sensu_stricto_1*, *Veillonella*, *Corynebacterium*, *Anaerococcus*, *Staphylococcus*, *Eubacterium*, *DTU089*, and *Eubacterium_brachy_group* showed significantly decreased abundance in the FGR group (Figure 3(e)). These results indicated alterations in the gut microbiota of FGR pregnant women.

### 3.4. Differences of Fecal Metabolites in FGR Pregnant Women

**Figure 4:**
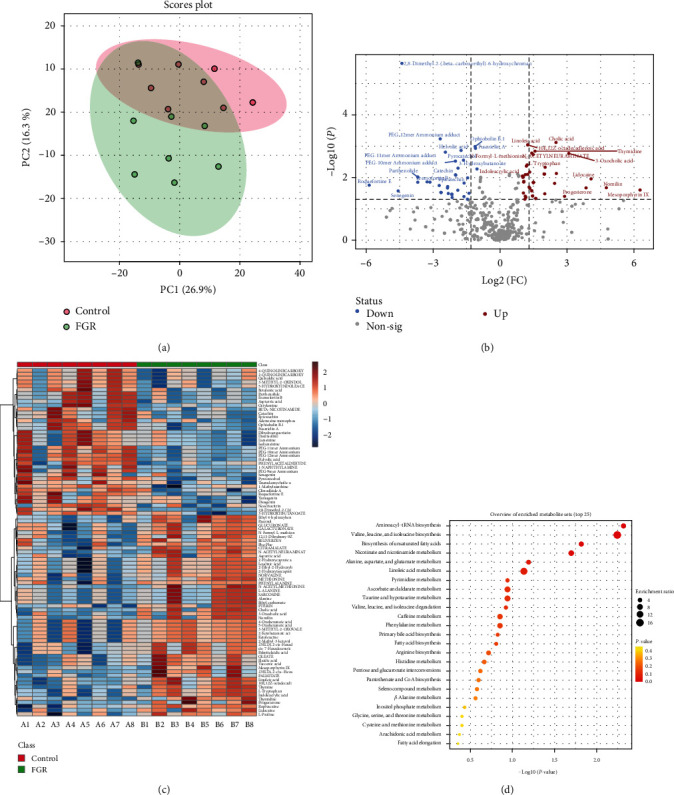
The difference of fecal metabolites in pregnant women with FGR: (a) PCA analysis, (b) volcano plot, (c) heatmap showed the differential metabolite abundance, and (d) KEGG function prediction showed the functional pathway of differential metabolite preference. *N* = 8 samples/group for the metabolomics analysis.

Differential metabolites were analyzed in fecal samples based on untargeted metabolomic analysis. PCA analysis showed overlapping and separation between the Control group and the FGR group, indicating changes in sample metabolism (Figure 4(a)). The volcano plot showed differential changes in 88 metabolites, with 37 downregulated and 51 upregulated metabolites (Figure 4(b)). The heatmap displayed the abundance changes of the 88 metabolites in the samples (Figure 4(c)). KEGG functional prediction revealed significant enrichment of the Aminoacyl-tRNA biosynthesis pathway, and Valine, leucine, and isoleucine biosynthesis pathway (Figure 4(d)). Among them, C00049 (Aspartic acid, up), C00073 (methionine, up), C00041 (alanine, up), and C00078 (L-tryptophan, up) showed differential changes in the Aminoacyl-tRNA biosynthesis pathway. C00671 (3-methyl-2-oxovalerate, up) and C00233 (Ketoleucine, up) showed differential changes in the valine, leucine, and isoleucine biosynthesis pathway (Figures 4(c) and 4(d)). These results demonstrated changes in the abundance and function of fecal metabolites in FGR pregnant women.

### 3.5. Correlation Analysis

**Figure 5:**
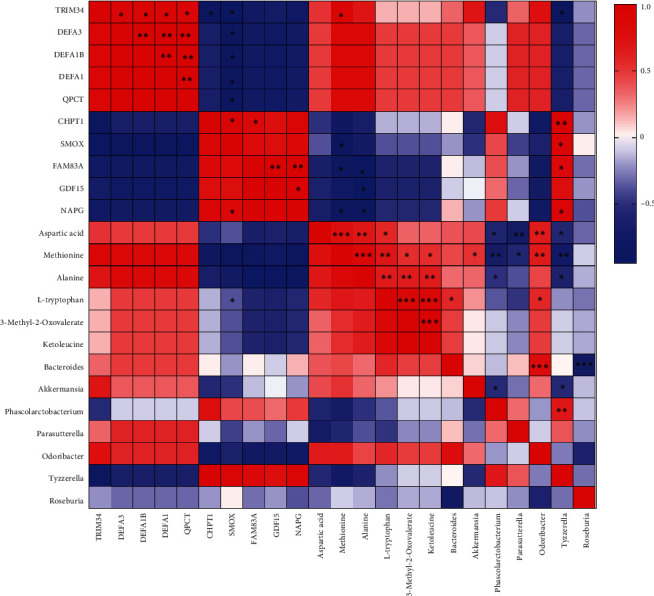
Correlation analysis of differentially circulating mRNA with microbiota and metabolites. Red represents positive correlation and blue represents negative correlation.  ^*∗*^*P* < 0.05,  ^*∗∗*^*P* < 0.01,  ^*∗∗∗*^*P* < 0.001.

We first analyzed the correlation between differential expression of mRNA and the microbiome and metabolites. The results showed that TRIM34 was positively correlated with DEFA3, DEFA1B, DEFA1, QPCT, and methionine, and negatively correlated with CHPT1, SMOX, and Tyzzerella (Figure 5). DEFA3, DEFA1B, DEFA1, and QPCT were positively correlated with TRIM34, DEFA3, DEFA1B, DEFA1, and QPCT, and negatively correlated with SMOX, respectively (Figure 5). CHPT1 was negatively correlated with TRIM34 and positively correlated with SMOX, FAM83A, and *Tyzzerella* (Figure 5). SMOX was negatively correlated with TRIM34, DEFA3, DEFA1B, DEFA1, QPCT, and methionine, and positively correlated with CHPT1 and *Tyzzerella* (Figure 5). FAM83A was negatively correlated with methionine and alanine, and positively correlated with CHPT1, GDF15, NAPG, and *Tyzzerella* (Figure 5). GDF15 was positively correlated with FAM83A and NAPG, and negatively correlated with alanine (Figure 5). NAPG was positively correlated with GDF15, FAM83A, and *Tyzzerella*, and negatively correlated with methionine and alanine (Figure 5).

Subsequently, we investigated the correlation between microbiota and metabolites. Among them, aspartic acid was positively correlated with methionine, alanine, L-tryptophan, and *Odoribacter*, and negatively correlated with *Phascolarctobacterium*, *Parasutterella*, and *Tyzzerella* (Figure 5). Methionine was positively correlated with aspartic acid, alanine, L-tryptophan, 3-methyl-2-oxovalerate, Ketoleucine, and *Odoribacter*, and negatively correlated with *Phascolarctobacterium*, *Parasutterella*, and *Tyzzerella* (Figure 5). Alanine was positively correlated with aspartic acid, methionine, L-tryptophan, and *Odoribacter*, and negatively correlated with *Phascolarctobacterium*, and *Tyzzerella* (Figure 5). L-tryptophan was positively correlated with aspartic acid, methionine, alanine, 3-methyl-2-oxovalerate, Ketoleucine, *Bacteroides*, and *Odoribacter* (Figure 5). Based on the above results, it can be observed that the metabolites (methionine, alanine), microbiota (*Tyzzerella*), and circulating mRNA (TRIM34, SMOX, FAM83A, NAPG) may serve as mediators in the interaction between the gut and circulatory system in FGR pregnant women.

## 4. Discussion

The occurrence of pre-eclampsia (PE) and FGR is linked to various overlapping differences in mRNA, lincRNA, and circRNA, while the consistency of small RNA differences is less significant [24]. Previous research indicates that identifying mRNA associated with growth genes in maternal blood can help detect severe preterm infants with FGR [25]. Furthermore, the expression of Flt-1 mRNA in maternal blood can act as an indicator for FGR and could predict its onset before clinical diagnosis [26]. RNA sequencing in this study verified that TRIM34, DEFA3, DEFA1B, DEFA1, and QPC were downregulated in umbilical cord blood, maternal serum, and placental tissue of newborns, while CHPT1, SMOX, FAM83A, GDF15, and NAPG were upregulated. These changes in gene expression might offer new markers for early screening of FGR and for further understanding its pathogenesis.

Placental dysfunction is a primary cause of FGR, which can often be effectively treated and managed with appropriate clinical intervention and attentive care [27]. Research indicates that the expression of GDF-15 mRNA and protein is increased in FGR-affected placentas, as well as in placentas affected by both FGR and PE [28]. The specific enrichment of glutamine and enhanced glutamine hydrolysis in individual natural killer (NK) cells is linked to increased trophoblast invasion and embryonic growth, facilitated through the secretion of insulin-like growth factor-1 and GDF-15 [29]. Therefore, a more in-depth investigation into the potential mechanisms and metabolic interactions involving upregulation of CHPT1, SMOX, FAM83A, GDF15, and NAPG in FGR could aid in the development of effective therapeutic approaches for treating FGR.

The relationship between maternal methionine amino acid metabolism disorder and its impact on fetal growth and development is becoming increasingly evident [30]. Some amino acids, such as alanine, valine, and isoleucine, are common metabolites in newborns and pregnant women [31]. The regulation of methionine amino acid metabolism during embryo and fetal development processes influences gene expression profiles and epigenetic characteristics of stem cells, promoting pluripotency and cellular function [32]. The kynurenines pathway, a metabolic intermediate of tryptophan, is active in the placenta during early pregnancy but downregulated in cases of hypoxia and FGR pregnancies [33]. Our study reveals significant functional changes in metabolites such as methionine, alanine, L-tryptophan, 3-methyl-2-oxovalerate, and ketoleucine. Methionine and alanine were found to be upregulated in cases of FGR and associated with alterations in circulating mRNA and microbial abundance, although their specific role in FGR remains unclear.

To further investigate the interaction mechanisms of mRNA, microbiota, and metabolites in FGR, we conducted correlation analysis to analyze the relationships of these factors. Among them, metabolites (methionine, alanine) as well as microbiota (*Tyzzerella*) and circulating mRNA (TRIM34, SMOX, FAM83A, NAPG) may serve as mediators communicating the interaction between the intestinal and circulatory systems of FGR pregnant women. *Tyzzerella* is one of the commensal bacteria in the human intestinal microbiota, such as *Tyzzerella nexilis* [34]. *Tyzzerella nexilis* can express the *β*-*n*-acetylhexosaminidase gene, which is involved in the synthesis of lacto-N-neotetraose (LNnT), serving as a precursor for human milk oligosaccharide synthesis [35]. The results of 16S rRNA sequencing showed a significant decrease in the abundance of *Tyzzerella* in the FGR group, and it was found to be correlated with various circulating mRNA and metabolites, suggesting a potential involvement in host human milk oligosaccharide synthesis. In addition, *Tyzzerella* and members of the Firmicutes phylum such as *Blautia*, *Clostridium*, *Enterococcus*, and *Ruminococcus* have the ability to produce a large amount of aromatic amines through the action of aromatic amino acid decarboxylase [36]. Therefore, further exploration of the role of *Tyzzerella* may be an effective means to improve FGR.

The alanine aminotransferase (ALT) levels and the abundance of *Tyzzerella* increase in patients with nonalcoholic fatty liver disease [37]. Feeding fish with soy protein concentrate (45%) increases ALT activity and enriches *Tyzzerella* and *Shewanella* of the Firmicutes and Proteobacteria phyla, showing slowed growth performance and poor health conditions [38]. Cooked adzuki beans can reduce ALT levels and intestinal *Tyzzerella* abundance in mitigating inflammation and metabolic disorders induced by a high-fat diet [39]. It is known that SMOX can mediate *β*-alanine metabolism [40], which is involved in metabolic disorders in offspring exposed to perfluorobutanesulfonic acid during pregnancy [41]. Our study shows a positive correlation between SMOX and *Tyzzerella*, which is negatively correlated with alanine. However, the role of SMOX-*Tyzzerella*-alanine metabolism in FGR is unknown and is one of the directions that needs to be further explored. Currently, except for GDF-15 and SMOX, there are no reports on the remaining eight mRNAs in pregnancy and FGR, which will be potential target genes for further investigation in FGR.

Preterm infants have a conservative and unique covarying microbiota in the early stages of their lives, which may have profound implications for their future development [42]. Additionally, during early and late pregnancy, the quantities of *Akkermansia*, *Bifidobacterium*, and *Firmicutes* increased, which may be related to the increased energy storage demand [43]. The abundance of *Bacteroides* and *Akkermansia* in the FGR group was also significantly increased, possibly in response to the energy demand of FGR pregnant women. However, the potential role of these microbiota in FGR still needs further investigation. Dietary supplementation of methionine increased the abundance of intestinal *Tyzzerella* and production of SCFA, alleviating oxidative stress and inflammatory responses in the hippocampus of d-galactose-induced aging mice [44]. A small randomized controlled trial demonstrated that supplementation with probiotics for two weeks after birth in newborns born at 35 weeks of gestation and who underwent one or more gastrointestinal surgeries resulted in significantly higher relative abundance of nonpathogenic gut bacteria (*Lactobacillaceae*) compared to infants receiving a placebo [45]. At present, there are few reports about other metabolites and microbiota in FGR, which need to be further explored. Therefore, further exploration of microbiota or metabolites related to FGR may help enhance nutritional management strategies for newborns affected by FGR.

In summary, this study confirms that the mRNA gene expression profile and function of umbilical cord blood in FGR pregnant women undergo changes. Among them, metabolites (methionine, alanine) as well as microbiota (*Tyzzerella*) and circulating mRNA (TRIM34, SMOX, FAM83A, NAPG) may serve as mediators communicating the interaction between the intestinal and circulatory systems of FGR pregnant women.

## Figures and Tables

**Table 1:** Clinical information of subjects.

Items	Control group (*n* = 11)	FGR group (*n* = 9)	*P*-value
Maternal age (year)	32.82 ± 4.09	30.33 ± 3.46	0.164
Maternal weight (kg)	69.71 ± 12.74	63.71 ± 10.56	0.273
Gestational age at delivery (weeks)	38.60 ± 1.66	37.22 ± 1.25	0.054
Birth weight (kg)	3.23 ± 0.55	2.27 ± 0.18	<0.001
Pregnant BMI (kg/m^2^)	21.03 ± 3.52	24.56 ± 10.92	0.797
Height (cm)	155.77 ± 4.74	155.33 ± 4.39	0.954
Pregnant weight (kg)	63.89 ± 9.83	62.63 ± 10.41	0.954
Weight gain (kg)	13.14 ± 3.00	12.17 ± 5.56	0.947
Fasting blood glucose (mmol/L)	5.22 ± 1.97	4.29 ± 0.34	0.716

**Table 2:** Primer sequence.

Gene	Sequence (5′−3′)	Length
H-CHPT1	F ATTGCGCTCATTGGCAGACT	190 bp
	R CCACCTAGAAATCCAAGAACTGG	—

H-SMOX	F GACTTACTTCCCCGGCTCAG	88 bp
	R CGCACTGTCACCACTGGATT	—

H-FAM83A	F CCTGTGATGGGCCTGAAGTC	175 bp
	R CATCTCAGCCGTCAGTTCGG	—

H-GDF15	F GCAAGAACTCAGGACGGTGA	146 bp
	R TGGAGTCTTCGGAGTGCAAC	—

H-NAPG	F TAAACGAGGGGCTGGAACAC	95 bp
	R TTCAGAAGCGGCACTGTCAT	—

H-TRIM34	F GCAACTTCTGCTTCAGCCATC	239 bp
	R CTGGTCACTGCCTCCTTGTT	—

H-DEFA3	F GCTAGAGGATCTGTGACCCC	204 bp
	R TGAGCCTGGATGCTTTGGAG	—

H-DEFA1B	F GCTAGAGGATCTGTGACCCC	172 bp
	R CCCATGCAAGGGAAACAACC	—

H-DEFA1	F GCTAGAGGATCTGTGACCCC	156 bp
	R AACCACTTCTGGGATGTCCG	—

H-QPCT	F GCTCCAAACCCAACGTTTCC	130 bp
	R GGAAATACCGCCCCTCCAAA	—

H-actin	F ACCCTGAAGTACCCCATCGAG	224 bp
	R AGCACAGCCTGGATAGCAAC	—

## Data Availability

The data used in this study are included in the paper. Raw data can be obtained from the corresponding author upon reasonable request.
